# Neurokinin-2 receptor negatively modulates substance P responses by forming complex with Neurokinin-1 receptor

**DOI:** 10.1186/s13578-023-01165-6

**Published:** 2023-11-15

**Authors:** Lan Phuong Nguyen, Minyeong Cho, Thai Uy Nguyen, Hee-Kyung Park, Huong Thi Nguyen, Kateryna Mykhailova, Sunghoon Hurh, Hong-Rae Kim, Jae Young Seong, Cheol Soon Lee, Byung-Joo Ham, Jong-Ik Hwang

**Affiliations:** 1 Department of Biomedical Sciences, College of Medicine, Korea University, Seoul, Republic of Korea; 2 Department of Psychiatry, College of Medicine, Korea University, Seoul, Republic of Korea

**Keywords:** Neurokinin receptors, GPCR dimerization, Negative modulation, Calcium signaling, ERK phosphorylation, β-arrestin, Cell migration

## Abstract

**Background:**

Tachykinins and their cognate receptors, neurokinin receptors (NKs) including NK1, NK2, and NK3 play vital roles in regulating various physiological processes including neurotransmission, nociception, inflammation, smooth muscle contractility, and stimulation of endocrine and exocrine gland secretion. Their abnormal expression has been reported to be associated with neurological disorders, inflammation, and cancer. Even though NKs are expressed in the same cells with their expression being inversely correlated in some conditions, there is no direct evidence to prove their interaction. Understanding the functional crosstalk between NKs in mediated downstream signaling and cellular responses may elucidate the roles of each receptor in pathophysiology.

**Results:**

In this study, we showed that NKs were co-expressed in some cells. However, different from NK3, which only forms homodimerization, we demonstrated a direct interaction between NK1 and NK2 at the protein level using co-immunoprecipitation and NanoBiT-based protein interaction analysis. Through heterodimerization, NK2 downregulated substance P-stimulated NK1 signals, such as intracellular Ca^2+^ mobilization and ERK phosphorylation, by enhancing β-arrestin recruitment, even at the ligand concentration that could not activate NK2 itself or in the presence of NK1 specific antagonist, aprepitant. In A549 cells with receptors deleted and reconstituted, NK2 exerted a negative effect on substance P/NK1-mediated cell migration.

**Conclusion:**

Our study has provided the first direct evidence of an interaction between NK1 and NK2, which highlights the functional relevance of their heterodimerization in cellular responses. Our findings demonstrated that through dimerization, NK2 exerts negative effects on downstream signaling and cellular response mediated by NK1. Moreover, this study has significant implications for understanding the complexity of GPCR dimerization and its effect on downstream signaling and cellular responses. Given the important roles of tachykinins and NKs in pathophysiology, these insights may provide clues for developing NKs-targeting drugs.

**Supplementary Information:**

The online version contains supplementary material available at 10.1186/s13578-023-01165-6.

## Background

The tachykinin family is a highly conserved group of multi-functional peptides, sharing a common C-terminal sequence (-Phe-X-Gly-Leu-Met-NH2, where X is a hydrophobic amino acid) [1]. In mammals, three genes encode three major tachykinins, *TAC1* (pre-pro-tachykinin-A, Ppt-a), *TAC3* (Ppt-b), and *TAC4* (Ppt-c). Alternative splicing of *TAC1* gives rise to four possible transcripts, including α-, β-, γ-, and δ-*TAC1* mRNAs, all of which are capable of producing substance P (SP). Neurokinin A (NKA) is generated by β- and γ-*TAC1* mRNAs, while neurokinin B (NKB) and hemokinin-1 (HK-1, SP-like tachykinin) are produced by proteolytic cleavage of *TAC3* and *TAC4* products, respectively [1, 2]. Tachykinins are widely expressed throughout neuronal and non-neuronal cells including immune cells where they regulate a wide range of physiological processes such as neurotransmission, nociception, inflammation, smooth muscle contractility, and stimulation of endocrine and exocrine gland secretion [3, 4].

Tachykinins exert their biological roles by binding to neurokinin receptors (NKs), which belong to class A of G-protein coupled receptors (GPCRs), specifically neurokinin 1 (NK1), neurokinin 2 (NK2), and neurokinin 3 (NK3). Although SP, NKA, and NKB preferentially bind to NK1, NK2, and NK3 receptors, respectively, they can activate other receptors with lower affinity [2, 5, 6]. Upon binding to tachykinins at the plasma membrane, NKs can couple with different Gα subunits (e.g., G_q/11_, G_s_, G_12/13_, G_i_), leading to activation of phospholipase C (PLC) and/or adenylate cyclase. Hydrolysis of phosphatidylinositol 4,5-biphosphate (PIP_2_) by PLC produces inositol (1,4,5)-trisphosphate (IP3) and diacylglycerol (DAG). IP3 can increase cytosolic Ca^2+^ concentration and DAG activates protein kinase C (PKC), which in turn can induce transcription of early genes (e.g., c-fos, c-myc) and activate mitogen-activated protein kinases (MAPK) including extracellular signal regulated kinases 1 and 2 (ERK1/2) [7–9]. Like most class A GPCRs, ligand stimulation leads to rapid desensitization and internalization of receptors mediated by GRKs phosphorylation and β-arrestin recruitment [10]. Notably, β-arrestin can also mediate NK1-stimulated cellular responses associated with sustained pain transmission or cancer cell proliferation [11, 12].

While tachykinin and their cognate receptors are widely expressed throughout the body, their distribution is uneven among different organs, tissues, and cell types [1, 4, 13]. For example, SP and NK1 are expressed widely in various regions such as nervous, cardiovascular, immune, and digestive system. Their involvement in different pathological processes such as neurological disorders, inflammation, and cancers has been widely reported [1, 3, 12]. Although NKA is expressed and released from the brain, NK2 is rarely found in adult human brain [1, 13]. However, their expression and roles are described in different cell lines such as immune cells and cancer cells. NKB and NK3 are expressed not only in the central nervous system, but also in the reproductive system. Thus, their roles are implicated in neurological diseases such as mood disorders, learning and memory deficiencies and reproduction-related disorders [14, 15]. Some studies have suggested a possible crosstalk between SP/NK1 and NKA/NK2, given the wide overlap in their distribution [16–20]. Among them, the study on primary human bone marrow stroma showed that increased expression of NK1 was correlated with decreased expression of NK2 at both mRNA and protein levels. Furthermore, the results demonstrated a yin-yang relationship between NK1 and NK2 in hematopoiesis, such that upregulation of NK1 enhanced the proliferation of bone marrow progenitors, while NK2 expression led to their growth inhibition [20].

Although some studies have reported that NK1 and NK2 are expressed in some cancers, the role of NK2 in cancer remains controversial. SP/NK1 has been shown to play a role in cancer progression by upregulating expression of anti-apoptotic genes and genes encoding matrix metalloproteinases (MMPs) [21]. In human colorectal cancer cell DLD-1, inhibition of SP/NK1 by FDA-approved NK1-specific antagonist aprepitant might lead to suppression of AKT/mTOR and Wnt/β-catenin signaling pathways, which play important roles in tumorigenesis [22]. On the other hand, NKA and NK2 have been reported to induce phosphorylation of ERK1/2 in the same cells. Furthermore, a selective NK2 antagonist not only reduced the viability and proliferation of DLD-1 cell, but also decreased tumor size in vivo [23]. However, other study has shown that NKA/NK2 can inhibit human leukemia cell proliferation and exert negative effects on NK1 [24]. In prostate cancer, NK1 and NK2 have been found to have an opposite effect on cancer progression [25, 26]. Overexpression of NK2 in cells originating from prostate cancers deactivates the Wnt/β-catenin signaling pathway, which might lead to inhibition cancer cell proliferation and migration. The anticancer effect of NK2 might be related to immune system modulation. A recent study has shown that the downregulation of NK2 expression can cause a remarkable increase in tumor growth in mice due to reduced infiltration of CD8 + T cells in tumor tissues [27]. Although NK1 and NK2 might be co-expressed in the same cells and functionally involved in the regulation of cancer progression, there is still a lack of direct evidence for the effect of their functional interaction on cellular responses. Moreover, most studies have been conducted using saturated and non-physiological concentrations of SP or NKA ranging from 10^–7^ to 10^–6^ M. These concentrations can even activate other NKs with full efficacy, making it hard to dissect precise roles of each receptor in pathophysiology.

In this study, we aimed to provide direct evidence for NK1-NK2 interaction using various assays including co-immunoprecipitation and NanoBiT technology. We also investigated the possible effect of this interaction on downstream signaling such as calcium mobilization, β-arrestin recruitment, and pERK1/2 level by comparing them to their individual signaling in cells expressing NK1 or NK2. Specifically, using human lung adenocarcinoma A549 cells endogenously expressing both receptors, we addressed the roles of individual receptors and their dimerization in mediating cell migration. Our data suggested that NK1 and NK2 dimerization might negatively modulate the function of NK1 in both signal transduction and cellular responses.

## Results

### Expression properties of NKs and their homo-/hetero-dimerization

**Fig. 1:**
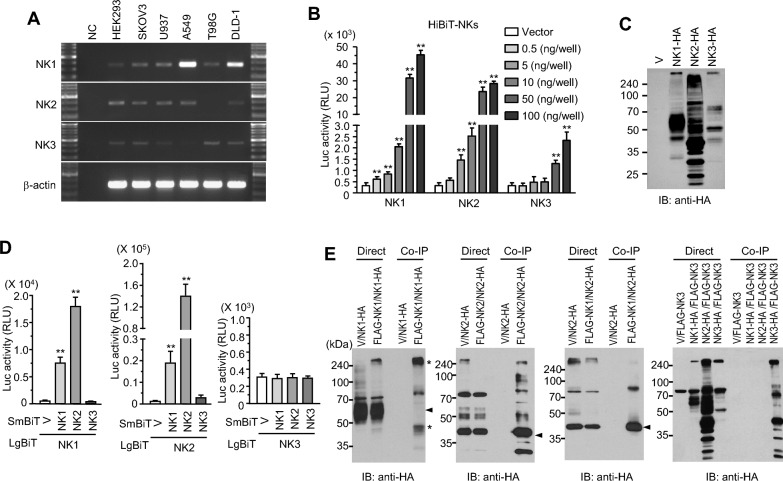
Expression properties and molecular interaction of neurokinin receptors (NKs). **A** RT-PCR analysis of mRNA expression. Total RNAs from cell lines were analyzed with isotype-specific primers and PCR products after 35 cycles for the receptors or 25 cycles for β-actin gene were visualized on a 1.5% agarose gel. A 1 kb-plus ladder was used as a size marker. **B** N-terminal HiBiT-tagged receptors were expressed in HEK293 cells with varying amounts of plasmids. HiBiT activity was assessed by adding Nano-Glo^®^ HiBiT extracellular detection reagent. **: *p* < 0.01 vs. vector **C** Expression of C-terminal HA-tagged NKs. HEK293 cells transfected with plasmids containing C-terminal HA-tagged NKs were lysed with RIPA buffer and subjected to western blotting with anti-HA antibodies. **D** Luminescence induced by receptor dimerization. HEK293 cells co-expressing NKs of C-terminal SmBiT- and LgBiT-tagged forms were analyzed by NanoBiT assay. **: *p* < 0.01 vs. vector (V). **E** Co-immunoprecipitation. HEK293 cells co-expressing FLAG- and HA-tagged receptors were lysed and subjected to immunoprecipitation with anti-FLAG agarose, followed by western blotting with anti-HA antibody

Individual NKs may play distinct roles in responses to their cognate neuropeptides. However, due to sequence similarity of peptides, these receptors are expected to affect each other, potentially provoking diverse cellular responses through ligand binding modes and receptor complex formation. As a first step to prove this idea, we investigated expression levels of these receptors in several cell lines. RT-PCR revealed that all three receptors were expressed in most cells with some exceptions, implying that they might have biological relevance to each other in cellular responses upon binding to ligands. According to their band intensities, NK1 might be the dominant receptor in A549 and DLD1 cells, whereas NK2 and NK3 might not be expressed in T98G and A549 cells, respectively (Fig. 1A). Next, their membrane localization was determined in cells expressing HiBiT-tagged forms since GPCRs function through neuropeptide binding on the cell surface. HiBiT-mediated luminescence increased depending on the plasmid amount transfected into HEK293 cells. Signals were quite strong in cells expressing NK1 and NK2, but relatively weak in NK3, implying that either protein expression or membrane localization of NK3 was relatively low (Fig. 1B). To determine protein amounts, cells expressing C-terminal HA-tagged receptors driven by CMV promoter were lysed with RIPA buffer and used for western blotting with anti-HA antibodies. As shown in Fig. 1C, the main band of NK1 was detected between 50 and 70 kDa. NK2 was detected as multiple bands, with the main signal located between 35 and 50 kDa. A clear band of NK3 was detected at 50 kDa, although there were weak bands at different sizes. Comparing total amounts of proteins, NK3 expression was prominently lower than those of others, indicating that NK3 was expressed with a low efficiency.

Next, complex formation of these receptors was investigated using NanoBiT. When C-terminal LgBiT-tagged NKs were expressed with C-terminal SmBiT-tagged NKs in HEK293 cells, luciferase activities were enhanced, indicating that LgBiT/SmBiT could bind through homo- or hetero-dimerization of NK1 and NK2. However, luciferase activities in cells expressing NK3 with either NK1 or NK2 were not increased, possibly due to a low expression of NK3 by a relatively weak Ubiquitin C promoter or structural incompatibility of NanoBiT-tagged form (Fig. 1D). Direct interaction between NK1 and NK2 was verified by co-immunoprecipitation. Due to migration properties of GPCRs on SDS-PAGE, precipitates were subjected to SDS-PAGE without boiling. Interestingly, co-precipitated NK1 was not detected at the size where directly loaded proteins were located. Instead, main precipitates were detected at the start line of the running gel. On the other hand, co-precipitated NK2 was detected at the same size as directly loaded ones, suggesting that NK2 could be easily dissociated from the protein complex by the sample buffer, whereas NK1 could not (Fig. 1E). We expected that NK3 might also bind to NK1 or NK2 since they belong to the same receptor group and share the same ligands. However, immunoprecipitation for NK3 revealed that NK3 itself was co-precipitated, whereas neither NK1 nor NK2 was precipitated, indicating that NK3 could homodimerize without heterodimerize with other members (see right blot in Fig. 1E). Based on these data, NK3 is expected to function independently without any influence from other receptors.

### Comparison of NK subtypes-mediated cellular responses using NanoBiT constructs

NKs have been extensively studied, especially with respect to neuropeptide-stimulated Ca^2+^ responses [1]. Recently, we developed a real-time calcium assay based on NanoBiT and the Ca^2+^-dependent interaction of calmodulin and its target proteins to analyze NK-mediated cellular responses [28]. First, neuropeptide-stimulated luciferase activation was measured in cells expressing NKs and calcium probes to determine the suitability of our assay method. Neuropeptides induced luciferase activation with slightly different patterns at 1 μM, indicating that all receptors could mediate cellular responses to neuropeptides at high concentration (Additional file 1: Fig. S1). Next, the activation efficiency of receptors by each peptide was investigated on Ca^2+^ responses based on NanoBiT assay. Dose–response curves revealed that all tested peptides acted as full agonists for the three NKs with different potency: SP > NKA = NKB for NK1; NKA >  > NKB > SP for NK2; NKB >  > NKA > SP for NK3. These preference results of tachykinin for each receptor were in agreement with previous reports showing that SP, NKA, and NKB were dominant ligands for NK1, NK2, and NK3, respectively (Additional file 1: Fig. S2A) [2, 4, 29].

Ligand-stimulated β-arrestin recruitment to receptors was also examined using the NanoBiT system. The NanoBiT system was used to create construct combinations of receptors and β-arrestin, which were tagged with either SmBiT or LgBiT at N- or C-terminal. These constructs were then expressed in HEK293 cells. The combination for each receptor showing the highest luciferase activity by ligand treatment was selected. Interaction of NK1-SmBiT with β-arrestin1-LgBiT was induced by all peptides in a dose-dependent manner, with SP being the dominant stimulator of this binding. The interaction of NK2-LgBiT with β-arrestin1-SmBiT was more sensitive to NKA, whereas it was barely induced by SP in terms of potency and efficacy (Additional file 1: Fig. S2B). Unfortunately, the interaction of NK3 with β-arrestin1 was difficult to determine with NanoBiT constructs due to a low expression level of the receptor. Overall, despite different sensitivity, both calcium assay and β-arrestin recruitment assay are feasible for analyzing ligand stimulation properties of GPCRs since they measure separated downstream signaling pathways.

### NK1 enhances SP-stimulated β-arrestin recruitment to NK2.

**Fig. 2:**
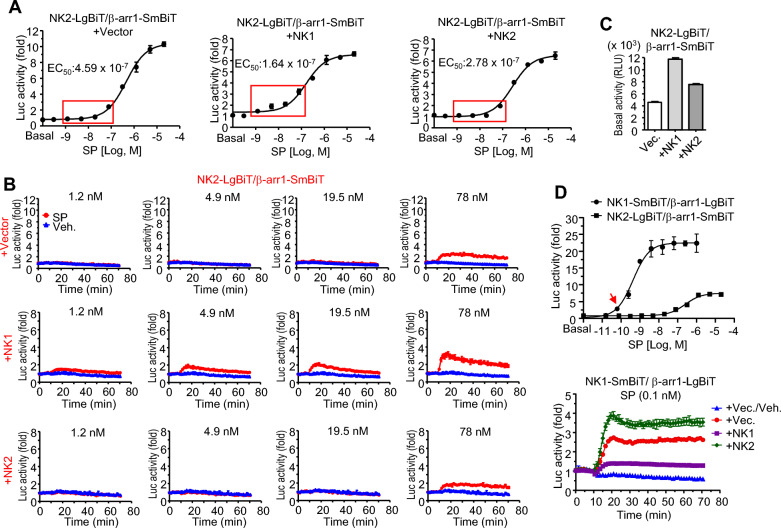
Effect of NK1 on SP-stimulated β-arrestin1 recruitment to NK2. **A** Cells expressing NK2-LgBiT and β-arrestin1-SmBiT with intact NKs were treated with different concentrations of SP and changes of luciferase activities were measured in real time. Maximal activities from each concentration were plotted on the graph. **B** Time-dependent responses at designated concentration which was boxed area in graphs of (A). **C** Basal luciferase activities in cells expressing NanoBiT constructs with intact receptors. **D** Effect of NKs on SP-stimulated β-arrestin1 recruitment to NK1. Upper graph: dose-dependent curves of SP-stimulated β-arrestin1 recruitment to NKs. Lower graph: luciferase activities of NK1-SmBiT and β-arrestin1-LgBiT in the presence of intact NKs in cells treated with 0.1 nM SP, a concentration that stimulated NK1 but not NK2 (indicated by red arrow in the upper graph)

Recruitment of β-arrestin1 to NKs clearly demonstrated that SP acted as a full agonist to NK1 but partial agonist to NK2 with relatively low affinity. Apart from properties of individual receptors, co-expression of multiple receptors in the same cells can result in complex formation and potentially lead to distinct signaling events compared to those mediated by individual receptors. Thus, dimerization effect of NK1 and NK2 was examined in SP-stimulated β-arrestin1 recruitment to receptors. We transfected HEK293 cells with three plasmids. Luciferase activities of NK2-LgBiT and β-arrestin1-SmBiT by SP were similar to those obtained from the system with two plasmids (459 nM vs. 409 nM in EC_50_) (Fig. 2A, left graph). Luciferase activities were slightly enhanced in the presence of intact NK1 (164 nM in EC_50_) (Fig. 2A, middle graph). However, luciferase activities were not changed by intact NK2 (278 nM in EC_50_) (Fig. 2A, right graph). The effect of NK1 on the interaction of NK2-LgBiT with β-arrestin1-SmBiT was obvious at a low concentration of SP (see red box). Figure 2B shows real-time luciferase activities by low concentrations of SP. The lowest concentration of SP to stimulate the luciferase activities through β-arrrestin1 interaction to NK2 was 78 nM, while activities were observed even at 1.2 nM SP in the presence of NK1, indicating that SP could stimulate NK1 and that ensuing GRK-dependent phosphorylation of both receptors in NK1/NK2-LgBiT complex was enough to recruit β-arrestin1-SmBiT to NK2-LgBiT (Fig. 2B). Basal luciferase activities of NK2-LgBiT and β-arrestin1-SmBiT were increased by NK1 compared to NK2, although the difference seemed to have no effect on ligand-stimulated activities (Fig. 2C). Dose-dependent curves of β-arrestin1 recruitment showed that NK1 was much more sensitive to SP than NK2. To explore the effect of NK2 on NK1/β-arrestin1 interaction, HEK293 cells expressing NK1-SmBiT and β-arrestin1-LgBiT with intact NKs were stimulated by 0.1 nM SP, a concentration that could induce β-arrestin1 recruitment to NK1-SmBiT but not to NK2-LgBiT. Co-expression of NK1 lowered luciferase activities of NK1-SmBiT/β-arrestin1-LgBiT by SP, implying that intact NK1 might compete with NK1-SmBiT for limited amount of ligands. In contrast, co-expression of NK2 enhanced luciferase activities, suggesting that NK1-SmBiT/NK2 complex might increase the chance to bind β-arrestin1-LgBiT, although NK2 itself could hardly bind to SP (Fig. 2D).

### NK2 negatively regulates NK1-mediated cellular responses to SP

**Fig. 3:**
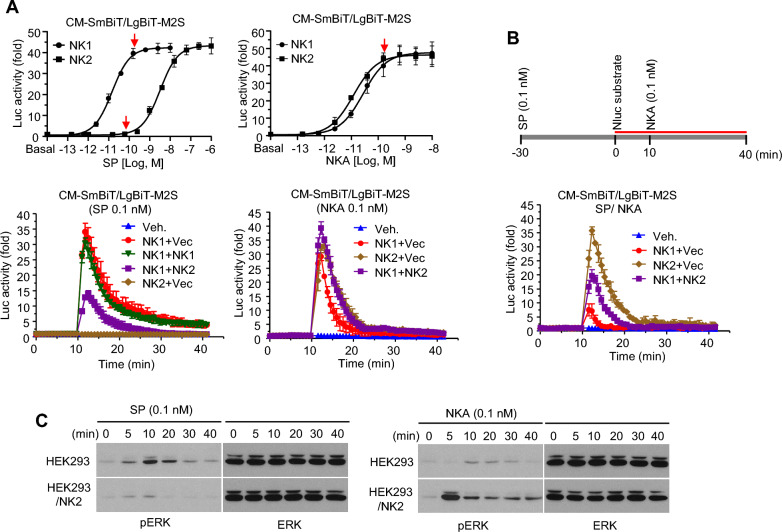
Effects of NK2 on NK1-mediated Ca^2+^ influx and ERK phosphorylation. **A** Optimal concentrations of tachykinin-stimulated Ca^2+^ responses were determined from dose–response curves. HEK293 cells expressing Ca^2+^ probes with NKs were treated with 0.1 nM SP or NKA. The concentration of SP at 0.1 nM was the concentration that fully stimulated NK1 but not NK2. The concentration of NK at 0.1 nM was the concentration that fully activated both receptors. **B** Effect of SP pretreatment on NKA-stimulated Ca^2+^ responses. Cells expressing Ca^2+^ probes with NKs were treated with 0.1 nM SP for 30 min. Medium was then replaced and 0.1 nM NKA was added. **C** HEK293 cells and exogenous NK2-expressing cells were serum-starved for 24 h and treated with 0.1 nM peptides. Cells were harvested at designated time point and subjected to western blotting with anti-ERK or phospho-ERK antibodies

Recruitment of β-arrestin to the cognate receptor upon ligand stimulation is an important step for downregulation of cellular responses to ligands by inducing receptor internalization. However, this process can also induce other signaling events apart from the ones mediated by heterotrimeric G proteins [30–32]. As previously mentioned, NK2 could enhance SP-stimulated β-arrestin interaction with NK1, suggesting a possible role of NK2 as a modulator of NK1 signaling. While NK1 was 100 folds more sensitive to SP than NK2 in terms of Ca^2+^ response (0.013 nM vs 2.2 nM in EC_50_, see Fig. S2A), both receptors had similar sensitivity to NKA (0.027 nM vs. 0.012 nM in EC_50_). To explore the effect of NK2 on NK1-mediated Ca^2+^ influx, cells expressing receptors with NanoBiT-based Ca^2+^ probes were treated with 0.1 nM SP, a concentration that induced nearly maximal activation of NK1 but no response of NK2. Transfection with different amounts of NK1 plasmids did not change the Ca^2+^ influx pattern possibly because this concentration of the ligand was almost at a saturation level. On the other hand, Ca^2+^ responses were significantly decreased in cells expressing both NK1 and NK2, although cells expressing NK2 alone did not show any response. This result suggests that NK2 might modulate NK1 activation through receptor complex formation (Fig. 3A, left graphs). Ca^2+^ responses to NKA were quite similar in cells expressing a single receptor or co-expressing both receptors since these receptors had similar sensitivity to NKA (Fig. 3A, right graph).

To further confirm the dimerization of NK1 and NK2, cells expressing receptors were pretreated with 0.1 nM SP for 30 min. After the medium was removed, NanoBiT substrate was added. Treatment with 0.1 nM NKA induced weak Ca^2+^ responses through a small number of NK1 proteins that remained unresponsive to SP. Cells expressing NK2 showed a strong Ca^2+^ response to NKA regardless of SP pretreatment. However, such response was significantly decreased in cells expressing both NK1 and NK2, suggesting that some NK2 proteins were dimerized with NK1 and internalized with their binding partners by SP stimulation. Consequently, the remaining NK2 attended to NKA binding and mediated Ca^2+^ response (Fig. 3B).

Most GPCRs can activate ERK phosphorylation, which is the most sensitive cellular response. When HEK293 cells were treated with 0.1 nM SP, ERK phosphorylation was transiently induced probably through NK1, implying that NK1 dominated in response to the ligand, although other NKs might be expressed according to RT-PCR. Interestingly, such ligand-stimulated phosphorylation was significantly reduced by exogenous expression of NK2. This might be another evidence of NK2’s ability to modulate SP-stimulated NK1 activation (Fig. 3C, left panel). NKA-dependent ERK phosphorylation was weakly induced in HEK293 cells. It was further increased in the presence of exogenous NK2, indicating that NK2 was a positive mediator of NKA (Fig. 3C, right panel).

### NK1-mediated NK2/β-arrestin1 interaction is inhibited by an NK1-specific inhibitor

**Fig. 4:**
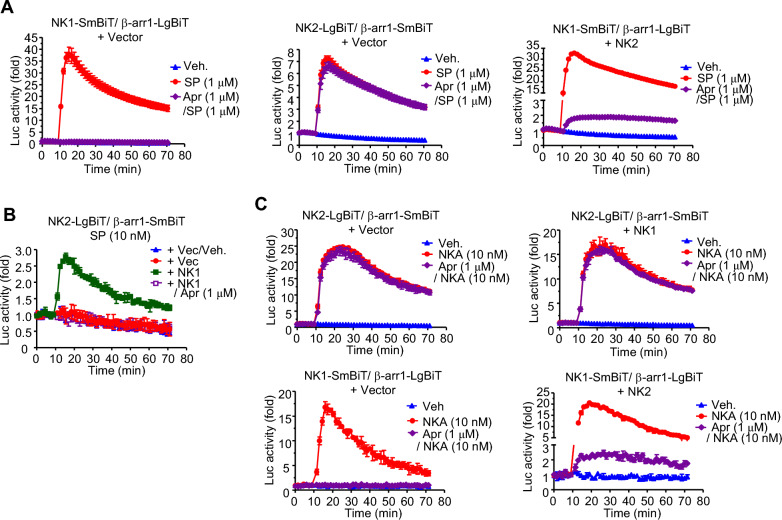
Inhibition of NK1-mediated NK2/β-arrestin1 interaction by NK1-specific inhibitor. **A** The effect of aprepitant on 1 μM SP-stimulated β-arrestin1 recruitment to NKs was examined in cells expressing NanoBiT constructs of NKs and β-arrestin1 with or without NK2. Cells were pretreated with 1 μM aprepitant for 30 min before the addition of SP. **B** Aprepitant inhibited 10 nM SP-stimulated interaction of NK2-LgBiT and β-arrestin1-SmBiT in the presence of NK1. **C** Aprepitant inhibited NKA-stimulated β-arrestin1 recruitment to NK1 but not NK2. Cells expressing NanoBiT constructs of NKs and β-arrestin1 with or without another NKs were pretreated with aprepitant for 30 min and then stimulated with 10 nM NKA. Luciferase activities were measured with a luminometer

The interaction between NK1 and NK2 was further confirmed using aprepitant, an NK1-specific inhibitor. SP-stimulated luciferase activities of NK1-SmBiT and β-arrestin1-LgBiT were completely inhibited by aprepitant even at a high concentration (1 μM), while luciferase activities of NK2-LgBiT and β-arrestin1-SmBiT were not affected by this inhibitor (Fig. 4A, left and middle graphs). However, when NanoBiT constructs of NK1 and β-arrestin1 were expressed with intact NK2, luciferase activities were slightly increased by SP even in the presence of the inhibitor (Fig. 4A, right graph). This result suggests that NK2 activated by high concentration of SP might recruit β-arrestin1-LgBiT to the complex of NK2 and NK1-SmBiT. Subsequently, β-arrestin1-LgBiT and inactive NK1-SmBiT caused by aprepitant were getting closer to each other enough to induce luciferase activation.

As another approach, cells expressing NK2-LgBiT and β-arrestin1-SmBiT with intact NK1 were treated with 10 nM SP, a non-effective concentration to activate NK2. Ligand-dependent luciferase activities were likely from NK1-dependent interaction of NK2-LgBiT and β-arrestin1-SmBiT, as aprepitant inhibited luciferase activities (Fig. 4B).

NKA-stimulated luciferase activities were not influenced by aprepitant in cells expressing both NK2-LgBiT and β-arrestin1-SmBiT (Fig. 4C, upper left). Luciferase activities were not downregulated by the inhibitor in cells expressing NanoBiT constructs with intact NK1 either, implying that inactive NK1 in the receptor complex had no effect on NKA-stimulated interaction of NK2-LgBiT with β-arrestin1-SmBiT (Fig. 4C, upper right). However, NKA-stimulated luciferase activities were inhibited by aprepitant in cells expressing both NK1-SmBiT and β-arrestin1-LgBiT, confirming that aprepitant was a specific inhibitor of NK1 (Fig. 4C, lower left). When intact NK2 was co-expressed with these NanoBiT constructs, NKA-stimulated luciferase activities were slightly increased by NKA in the presence of aprepitant, similar to the response to SP in cells expressing the same plasmids. This result indicates NK2-dependent interaction of inactive NK1-SmBiT and β-arrestin1-LgBiT (Fig. 4C, lower right).

### NK1-mediated responses to SP are downregulated by NK2 in A549 cells

Based on results of RT-PCR (Fig. 1A), A549 cells are expected to express both NK1 and NK2. To confirm the expression of these two receptors, we measured ligand-stimulated ERK phosphorylation. Although basal levels of phospho-ERK were high in 24 h serum-starved A549 cells, treatment with 0.1 nM SP induced ERK phosphorylation, indicating expression of NK1 proteins in cells. Furthermore, exogenous expression of NK2 led to a decrease in SP-stimulated ERK phosphorylation, while 0.1 nM NKA slightly increased ERK phosphorylation and significantly increased it in the presence of exogenous NK2 (Additional file 1: Fig. S3).

**Fig. 5:**
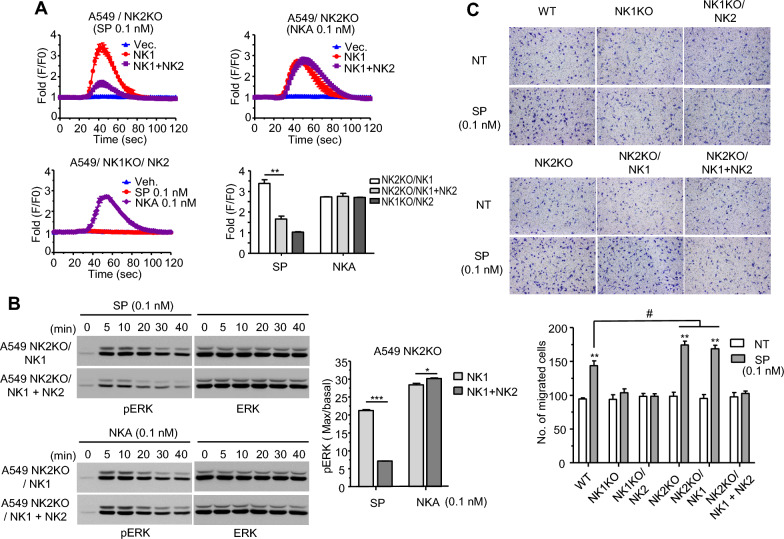
NK1-mediated responses to SP were downregulated by NK2 in A549 cells.** A** A549 cells lacking either NK were reconstituted with exogenous NKs. After incubation with calcium 6 reagent, cells were treated with 0.1 nM SP or NKA and the calcium responses were measured with a FlexStation^®^ 3 microplate reader. Maximal increases are shown in the graph. **: *p* < 0.01 (NK1 alone vs. NK1 + NK2). **B** A549 cells lacking NK2 and reconstituted with NKs were starved for 24 h and treated with 0.1 nM SP or NKA. Cells were lysed at the designated time point and cell lysates were subjected to western blotting with anti-ERK or phospho-ERK antibodies. The graph shows the intensity of maximal phosphorylation normalized with ERK. *: *p* < 0.05, ***: p < 0.001 (NK1 alone vs. NK1 + NK2). **C** Migration of A549 cells toward 0.1 nM SP. Cells lacking NKs and reconstituted with NKs in pictures were added into upper wells of transwell migration chambers and 0.1 nM SP was added to the lower well. After 18 h of incubation, non-migrated cells in upper well were removed with a cotton swab and cells migrated to the lower surface of the transwell were stained with Diff-quick staining solution and counted under a microscope. **: *p* < 0.01 vs. not treated. #: *p* < 0.05 (wild type vs. NK2KO or NK2KO + NK1)

To further investigate the effect of NK2 on SP-stimulated NK1 signaling, we generated A549 cells lacking either NK1 or NK2 using CRISPR/Cas9. These cells did not respond to low concentrations of tachykinins with respect to Ca^2+^ responses. When NK1 was exogenously expressed in A549 cells, 0.1 nM SP increased Ca^2+^ influx, which was significantly decreased by co-expression of NK1 and NK2 (Fig. 5A, upper left). However, the Ca^2+^ influx patterns by 0.1 nM NKA were quite the same for cells expressing NK1 with or without NK2 (Fig. 5A, upper right). When NK2 was exogenously expressed in NK1 knock-out A549 cells, NKA induced Ca^2+^ influx, but SP did not (Fig. 5A, lower left). The SP-stimulated maximal Ca^2+^ increase through NK1 was significantly downregulated by NK2 expression, although maximal responses to NKA were similar under all conditions (Fig. 5A, lower right). In NK2 knock-out A549 cells expressing exogenous NK1, treatment with 0.1 nM SP resulted in prominent and sustained ERK phosphorylation, while phosphorylation intensity was decreased in cells co-expressing NK1 and NK2, indicating negative modulation of NK1 by NK2. However, 0.1 nM NKA-stimulated ERK phosphorylation was quite similar under all expression conditions (Fig. 5B).

Furthermore, since SP has been reported to enhance cellular migration [8, 25, 33, 34], we performed a chemotactic assay with NKs-deleted and reconstituted A549 cells in the presence of the ligand. A549 cells were active in movement. Their migration was enhanced by 0.1 nM SP. However, migration was not increased in NK1 knock-out cells with or without exogenous NK2. Interestingly, NK2 knock-out cells migrated well regardless of exogenous NK1, while NK2 reconstitution reduced migration, suggesting that NK2 could negatively modulate NK1-mediated cellular migration toward SP.

## Discussion

The classical model of GPCRs pharmacology assumed that they exist and act as monomers. However, emerging evidence over the last two decades suggests that GPCRs might form and function as homo- and heterodimers or higher order oligomeric complexes with homologous receptor subtypes activated by the same endogenous agonists or with distantly related receptors. Complex formation can modulate receptor function by affecting coupling efficacy and preference to subtypes of heterotrimeric G proteins or β-arrestin and intracellular trafficking [35]. Several studies have reported that crosstalk among receptors can enhance signaling. For example, dimerization between muscarinic M2 and M3 receptors can lead to synergistic activation in secondary messengers [36]. Synergism in ERK1/2 activation was observed in cells co-expressing α1A and α1B adrenergic receptors in the presence of their cognate ligands [37]. On the other hand, some reports have indicated that receptor association might bring about negative modulation in downstream signaling [38]. One of the most well-studied examples is the heterodimer between µ-opioid and α2A adrenergic receptors. Morphine and noradrenaline are endogenous ligands of µ-opioid and α2A adrenergic receptors, respectively. Co-expression of these two receptors could lead to down-regulation of ERK1/2 phosphorylation mediated by noradrenaline in the presence of morphine [39]. Given their roles in physiological and pathological processes and their potential to alter pharmacologic properties, GPCR dimers might be unprecedented new targets for drug discovery.

To explore homo- or hetero-dimerization of NKs, we utilized protein–protein interaction (PPI)-based assays such as NanoBiT and co-immunoprecipitation. These assays take advantage of appropriate expression of target proteins in cells. We found that NK1 and NK2 were likely expressed well and localized to the cell surface where they would function. Although we were able to characterize functional properties of NK3 in terms of tachykinin stimulated Ca^2+^ responses, total protein level and membrane expression of NK3 were too low to attempt these NanoBiT-based assays. Furthermore, coimmunoprecipitation revealed that NK3 did not interact with NK1 or NK2. This finding in agreement with previous report [19], suggesting that NKB/NK3 tends to function independently. For this reason, we focused our analysis on crosstalk between NK1 and NK2 with their cognate ligands.

As previously mentioned, many cell-based assays to characterize NKs have been conducted with relatively high concentrations of neuropeptides, although their activation could be stimulated by authentic ligands at sub-nanomolar concentrations [29, 40–45]. Dose–response curves of each receptor to tachykinins in Ca^2+^ influx revealed that all receptors were activated by the three peptides with different potencies but quite similar efficacies. Therefore, high doses of peptides can activate all NKs expressed in the same cells, leading to saturated cellular responses. This makes it difficult to distinguish each ligand or receptor-specific response and study functional properties of molecular interaction between receptors. Our Ca^2+^ data also revealed much lower EC_50_ values for all tested peptides compared to previous studies using chemical synthesized Ca^2+^ indicators, suggesting that NanoBiT-based cytosolic Ca^2+^ assay is more sensitive and precise in characterizing GPCR-mediated Ca^2+^ signaling [46–48]. Furthermore, our Ca^2+^ assay seems to be useful for investigating the effect of receptor dimerization on cellular responses. When both NK1 and NK2 were expressed in the same cells, NK1-mediated Ca^2+^ influx was significantly decreased in response to 0.1 nM SP, a concentration that could not activate NK2. ERK phosphorylation is the most sensitive cellular response to ligand-stimulated GPCR activation. SP induced phosphorylation but not Ca^2+^ influx without exogenous NK1 expression in HEK293 cells. This signaling event might also confirm endogenous NK1 expression previously identified by RT-PCR. However, SP-dependent ERK phosphorylation was prominently decreased by exogenous NK2 expression.

In this study, we aimed to explore effects of receptor dimerization at physiological concentration of ligands using appropriate methodological approaches. Our previous reports have demonstrated that dimerization of GPCRs can be verified by coimmunoprecipitation as well as NanoBiT assay, which allows us to assess spatiotemporal interaction of proteins. We observed that NK2 responded to relatively high concentrations of SP in comparison to NK1 based on dose–response curves for Ca^2+^ influx and β-arrestin recruitment. However, in the presence of NK1, SP-stimulated β-arrestin1-SmBiT interaction to NK2-LgBiT occurred even at a low concentration of SP that could not induce β-arrestin recruitment to NK2. Interestingly, the interaction of β-arrestin1-LgBiT with NK1-SmBiT induced by 0.1 nM SP was declined in the presence of intact NK1 but enhanced in the presence of intact NK2. This phenomenon is likely due to the absolute number of responding receptors to the ligand, which is affected by receptor dimerization. If receptors exist as monomers and homo-/hetero-dimers, there might be several forms of receptors in cells, including NK1, NK1-SmBiT, NK1/NK1, NK1/NK1-SmBiT, and NK1-SmBiT/NK1-SmBiT. NK1 and NK1/NK1 might compete with SmBiT-tagged forms on SP binding, thereby reducing luciferase activities from the interaction of NK1-SmBiT with β-arrestin1-LgBiT. However, co-expression of NK2 might enhance the number of SmBiT-tagged forms such as NK1-SmBiT, NK1-SmBiT/NK1-SmBiT, and NK2/NK1-SmBiT which can recruit β-arrestin1-LgBiT by responding to SP, thereby enhancing luciferase activities. Although β-arrestins are responsible for internalization and desensitization of ligand occupied receptors, they also serve as scaffolds and adapters to mediate and prolong SP/NK1 signaling, which is involved in different diseases such as chronic pain and cancer proliferation [11, 49]. Our data revealed that NK2 might lead to β-arrestin recruitment to the complex of NK1 and NK2 at a high concentration of SP, even in the presence of aprepitant, a NK1-specific antagonist. Thus, we speculated that dimerization between NK1 and NK2 might partly explain limited application of aprepitant in clinical trials, although the compound has been proved to possess antitumor activities through promoting apoptotic mechanisms [50]. However, further studies are warranted to investigate the internalization and location of this complex in the endosome and its possible effect on NK1 endosomal signaling.

Tachykinins and their receptors play important roles in various physiological processes. While interactions between ligands and receptors are important, the specific receptor involved can have a significant impact on cellular responses. This is particularly true given the promiscuity of ligand/receptor interactions, especially for NK1 which has high affinity for all three tachykinins, despite SP being considered the authentic ligand. NK1 has been implicated as a key mediator of inflammation and cancer progression due to its upregulation in these pathological conditions [12, 21], while NK2 seems to play a negative modulatory role. In this study, we investigated roles of these receptors in A549 lung cancer cells through gene deletion and reconstitution. Our results showed that NK2 negatively modulated SP-stimulated Ca^2+^ response and ERK phosphorylation in these cells. Although SP has been reported to act as universal mitogenic compound in various cancer cells [25, 34], we did not observe the effect of SP on A549 cell proliferation in serum-free condition even at a high concentration (data not shown) in agreement with previous study [51]; however, it did stimulate chemotactic activity, which was found to be NK1-dependent. Interestingly, we found that exogenous NK2 decreased SP-stimulated migration, suggesting that NK2 was a negative modulator of SP-stimulated migration. Taken together, our findings demonstrate a direct interaction between NK1 and NK2 on the cell surface, whose formation may facilitate SP-dependent NK1 internalization by enhancing β-arrestin recruitment. Through NK1 interaction, NK2 is likely to play a role as a negative modulator of SP to affect cellular responses, especially migration and metastatic activity of cancer cells. Finally, our study highlights the importance of using physiological concentrations of ligands to understand functional properties of the ligand/receptor in cellular responses and related pathological conditions.

## Conclusion

Although the three members of the neurokinin receptors (NKs) appear to be expressed in most cell lines at transcription level, their preferences in forming receptor complex are different. The data showed that NK3 tends to form homodimers, suggesting its ability to function independently. On the other hand, NK1 and NK2 appear to interact with each other. Through heterodimerization, NK2 downregulates signaling mediated by SP/NK1, such as Ca^2+^ mobilization and ERK phosphorylation, by enhancing β-arrestin recruitment. This interaction further leads to negative modulation of the chemotactic effect of SP in cancer cells. Our study highlighted the complexity of GPCR dimerization and its impact on downstream signaling and cellular responses. Given the important roles of tachykinins and NKs in pathophysiology, these insights may provide valuable information for the development of drugs targeting NKs.

## Materials and methods

### Materials

SP (cat. no. S6883), NKA (cat. no. N4267) and NKB (cat. no. N4143) were acquired from Sigma-Aldrich (St. Louis, MO, USA) and dissolved according to the manufacturer’s recommendation. NanoBiT and HiBiT starter kits were purchased from Promega (Madison, WI, USA), containing plasmids and all necessary reagents for protein interaction assay and membrane protein expression assay, respectively. The pcDNA3.1 expression vector was provided by Invitrogen (San Diego, CA, USA). All PCR primers and related reagents were obtained from Cosmo Genetech (Seoul, Korea). DNA sequencing was conducted by Macrogen (Seoul, Korea). Primary antibodies such as anti-HA antibody (cat. no. ab9110) from Abcam (Cambridge, UK), agarose beads conjugated with anti-FLAG antibody (cat. no. A2220) from Sigma-Aldrich (St. Louis, Mo, USA), anti-ERK1/2 (cat. no. sc-514302) from Santa Cruz Biotechnogy (Dallas, Texas, USA), and anti-pERK1/2 (Thr202/Tyr204) (cat. no. 4370) from Cell Signaling Technology (Danvers, MA, USA) were used. Anti-rabbit and anti-mouse secondary antibodies were purchased from SeraCare (Milford, MA, USA).

### Cell culture

HEK293 and A549 cells were obtained from the American Type Culture Collection (ATCC, Manassas, VA, USA) and maintained in Dulbecco’s modified Eagle medium (DMEM) and RPMI, respectively. Both media were supplemented with 10% heat-inactivated fetal bovine serum (FBS), 100 U/ml penicillin G, and 100 µg/ml streptomycin. These cells were cultured in a humidified CO_2_ incubator at 37 °C.

### Plasmid construction

The CMV promoter sequence in pcDNA3.1 vector was substituted with promoters from Ubiquitin C gene (UbiC) to optimize expression levels of all proteins used for NanoBiT and HiBiT assays. HA- or FLAG-tagged receptors were expressed under control of CMV promoter in pcDNA3.1.

### RT-PCR

The day before RNA extraction, cells were seeded in a 60-mm cultured dish. Total RNA was extracted using TRIzol (Invitrogen) according to the manufacturer’s instruction and 3 µg of RNA was reverse transcribed using M-MLV reverse transcriptase (Promega, Madison, WI, USA) to synthesize cDNAs. cDNAs were then tenfold diluted and subjected to amplification using 25 or 35 cycles of 95 ℃ for 15 s, 57 ℃ for 30 s, and 72 ℃ for 30 s. The following sequence-specific primers were used for each gene: NK1 (461 bp), F: TGAAATCCACCCGGTATCTCC and R: TTCCCTAACCCATACTGACC; NK2 (374 bp), F: GCCCTACCACCTCTACTTCAT and R: AGCAAACCATACCCAAACCA; NK3 (425 bp), F: GCAGCAGAAACCTGGATAGA and R: AGCGCGTAGATGAAATTGAC; β-actin (313 bp), F: CACTCTTCCAGCCTTCCTTC and R: CTCGTCATACTCCTGCTTGC. PCR products were separated by 1.5% agarose gel electrophoresis.

### Western blotting and co-immunoprecipitation

Cells were lysed using RIPA buffer (150 mM NaCl, 50 mM Tris–HCl pH 8.0, 1% Triton X-100, 0.5% Sodium deoxycholate, 0.1% SDS, 20 mM NaF) and protease inhibitor cocktail (Roche, Indianapolis, IN, USA). Protein quantification was carried out using a Bradford protein assay kit (Bio-rad, Hercules, CA, USA). Protein extracts were clarified by centrifugation at 15,000 rpm for 15 min at 4 ℃. Subsequently, 10 µg of each sample was loaded and separated by 10% SDS-PAGE and transferred onto nitrocellulose membranes. These membranes were then blocked with 5% skimmed milk in Tris-buffered saline with Tween 20 for 30 min and incubated with specific primary antibodies (anti-ERK1/2 and anti-pERK1/2, 1:2000 dilution; anti-HA, 1:10000 dilution) overnight at 4, followed by incubation with HRP-labeled secondary antibodies (1:5000 dilution) for 1 h at room temperature. Protein bands were detected using an enhanced chemiluminescence kit (Thermo Fisher Scientific, Rockford, IL, USA). The relative band intensity of each blot was visualized and analyzed with E-blot (Shanghai, China).

To detect receptor homodimerization or heterodimerization, co-immunoprecipitation was performed. Cells were seeded into 60-mm dishes and transfected with plasmids containing either HA- or FLAG-tagged receptor genes. At 36 h after transfection, cells were washed with cold phosphate-buffered saline (PBS) and lysed with 1 ml lysis buffer (150 mM NaCl, 50 mM Tris–HCl, pH 7.5, 10 mM KCl, 1% Triton-X100, 10 mM NaF) for 30 min. Supernatants were collected after centrifugation at 15,000 rpm for 15 min at 4 ℃ and incubated with agarose beads conjugated with anti-FLAG antibodies at 4 ℃ for 2 h. Beads were then washed briefly with lysis buffer five times. Precipitates were separated by 10% SDS-PAGE and blotted with anti-HA antibodies.

### HiBiT assay for membrane expression of NKs

Membrane expression of NKs was detected using the Nano-Glo^®^ HiBiT extracellular detection system. HEK293 cells were transiently transfected with different amounts (0.5, 5, 10, 50, 100 ng/well) of plasmids containing each of N-terminal HiBiT-tagged receptors. At 24 h after transfection, the medium was replaced with 100 µl of serum-free medium and the plate was equilibrated at room temperature for 5 min. Next, 100 µl of Nano-Glo^®^ HiBiT extracellular detection system, which contained 1 µl of LgBiT proteins, 2 µl of Nano-Glo HiBiT substrate, and 97 µl of Nano-Glo HiBiT buffer, was added to each well. The plate was incubated at room temperature for 4 min before being measured with a luminometer (BioTek, USA).

### NanoBiT Complementation assay

NanoLuc Binary Technology (NanoBiT) was utilized to investigate protein–protein interaction. HEK293 cells were seeded in a 96-well white plate at a density of 2 × 10^4^ cells per well. The following day, cells were transfected with 50 ng of each plasmid containing SmBiT- or LgBiT- tagged genes using 0.2 µl Lipofectamine 2000 (Invitrogen, Carlsbad, CA, USA). To investigate the effect of one receptor on another receptor in β-arrestin recruitment, 100 ng of un-tagged receptor was co-transfected with 50 ng of each of NanoBiT-tagged plasmid and mixed with 0.4 µl Lipofectamine 2000. At 24 h after transfection, the medium was changed to 100 µl of Opti-MEM and the plate was stabilized at room temperature for 10 min before adding NanoBiT substrate. If an inhibitor was used, it was pre-incubated at 37 ℃ for 20 min and at room temperature for 10 min before substrate addition. Baseline luminescence was measure for 10 min using a SpectraMax L plate reader (Molecular Devices, San Jose, CA, USA). After stimulation with ligands, the luminescence signal was continuously measured for 1 h.

### Detection of intracellular calcium increase

Cytosolic Ca^2+^ changes were detected using a NanoBiT system as described previously [28]. In short, HEK293 cells were transfected with a total of 90 ng/well of plasmids, including 30 ng each of untagged receptor, calmodulin (CM) tagged with SmBiT at C-terminal, and the binding motif of myosin-light chain kinase 2 (M2S) tagged with LgBiT. In case of co-expressing the receptor, an additional 30 ng of each untagged receptor was used, resulting in a total amount of transfected plasmids of 120 ng/well. The measurement of changes in luminescence signal was similar to the description provided above.

Changes in cytosolic Ca^2+^ were examined in A549 knockout (KO) and A549 cells stably expressing NK1 or NK2 using a FLIPR Calcium 6 kit (Molecular Devices, San Jose, CA, USA). Cells were seeded into 96-well black wall and clear bottom plates at density of 3 × 10^4^ cells per well. The following day, cells were loaded with FLIPR Calcium 6 dye mixed at a 1:1 ratio with medium supplied with 2.5 mM probenecid. The assay plate was wrapped with aluminum foil and incubated at 37 ℃ for 2 h in a humidified CO_2_ incubator. To improve ligand stability, different concentrations of SP and NKA were dissolved in 0.01% fatty acid free BSA and then automatically injected into the assay plate. Subsequently, changes in fluorescence intensity (excitation at 485 nm, emission at 525 nm) were recorded using a FlexStation 3 multi-mode microplate reader (Molecular Devices, San Jose, CA, USA).

### Establishment of knockout cells by CRISPR-Cas9

To establish cells lacking NK1 or NK2 expression, four potential target sequences were selected using a guide design program from the Zhang Lab (https://zlab.bio/guide-design-resources). A set consisting of forward and reverse strand oligos for target sequences were annealed and inserted into the pRG2 vector to express guide RNAs. Next, a 49-nucleotide sequence including the target site and adjacent sequences was inserted into the pMRS surrogate vector, which was then transfected into HEK293 cell together with p3S-Cas9 plasmids. The guide efficiency was assessed by genomic DNA PCR with appropriate primers and T7E1 treatment. Efficient guide vectors were chosen as follows: NK1: 5’-AGCTGCCTACACGGTCATTGTGG-3’; NK2: 5’-CAGGATGATCCAGATGACGATGG-3’. The surrogate vector and p3S-Cas9 with a guide vector were transfected into A549 cells. Potential KO cells were isolated using MACSelect Kk MicroBeads (Miltenyi Biotec, Bergisch Gladbach, Germany) and transferred into 96-well plates at a density of 0.5 cell/well. NK1 KO and NK2 KO were confirmed by genomic DNA PCR. To characterize individual signaling, NK1 was stably expressed in NK2 KO cells, while NK2 was stably expressed in NK1 KO cells.

### Transwell migration assay

A549 cells suspended in 100 µl RPMI with 0.1% FBS were seeded at a density of 1 × 10^4^ cells per well into the upper chamber of an 8-µm pore size transwell plate (Corning Inc. Corning, NY, USA). Next, 650 µl of RPMI containing 0.1% FBS with or without 0.1 nM SP was added to the lower chamber. The plate was kept at 37 °C in a humidified CO_2_ incubator. After 24 h, non-migrated cells in the upper chamber were removed using a wet cotton swab. Cells that had migrated through the filter were fixed with methanol, stained with hematoxylin and eosin, and then counted from randomly chosen optical microscopic field (100 × objective).

### Statistical analysis

All data are presented as mean ± standard deviation (SD) calculated by a GraphPad Prism 5 software (San Diego, CA, USA). Statistical significance was calculated using the unpaired *t*-test for comparison of two groups or one-way ANOVA with Bonferroni’s tests for comparison of three or more groups. A *p-*value less than 0.05 (*p* < 0.05) was considered statistically significant. All experiments were independently performed at least three times unless otherwise indicated.

### Supplementary Information

**Additional file 1: Fig. S1 **Ca^2+^ responses mediated by all neurokinin receptors (NKs) to 1 μM tachykinins. HEK293 cells expressing Ca^2+^ probes with each NK were treated with 1 μM of SP, NKA, and NKB. Luciferase activities were measured with a luminometer. **Fig. S2 **Dose responses of NKs to each tachykinin. **A** HEK293 cells expressing Ca^2+^ probes with each NK were treated with serially dilute ed ligands and luciferase activities were measured. **B** Cells expressing NanoBiT constructs of β-arrestin1 and NKs were treated with serially diluted ligands. Their EC_50_ values were designated in the tables. **Fig. S3 **The effect of exogenous NK2 on tachykinin-stimulated ERK phosphorylation. Parental A549 cells and exogenous NK2-expressing cells were incubated with serum-free media for 24 h and treated with 0.1 nM tachykinins for designated time. After lysis the cell extracts were applied to western blotting with anti-ERK and anti-pERK antibodies. The graph shows maximal pERK levels in comparison to basal levels, which were normalized with ERK blots. ***: p<0.001 vs maximal pERK in parental cells.

## Data Availability

Please contact the corresponding author for data, which will be provided on reasonable request

## Abbreviations

NKs: Neurokinin receptors
NK1: Neurokinin-1 receptor
NK2: Neuroknin-2 receptor
NK3: Neurokinin-3 receptor
GPCR: G protein-coupled receptor
SP: Substance P
NKA: Neurokinin A
NKB: Neurokinin B
NanoBiT: NanoLuc Binary Technology
SmBiT: Small BiT
LgBiT: Large BiT
CM: Calmodulin
M2S: Binding motif of myosin-light chain kinase 2
