# Supplementary material for: Neurokinin-2 receptor negatively modulates substance P responses by forming complex with Neurokinin-1 receptor
Source: Cell Biosci. 2023 Nov 15;13:212. doi: 10.1186/s13578-023-01165-6 (PMC10652611; doi:10.1186/s13578-023-01165-6)

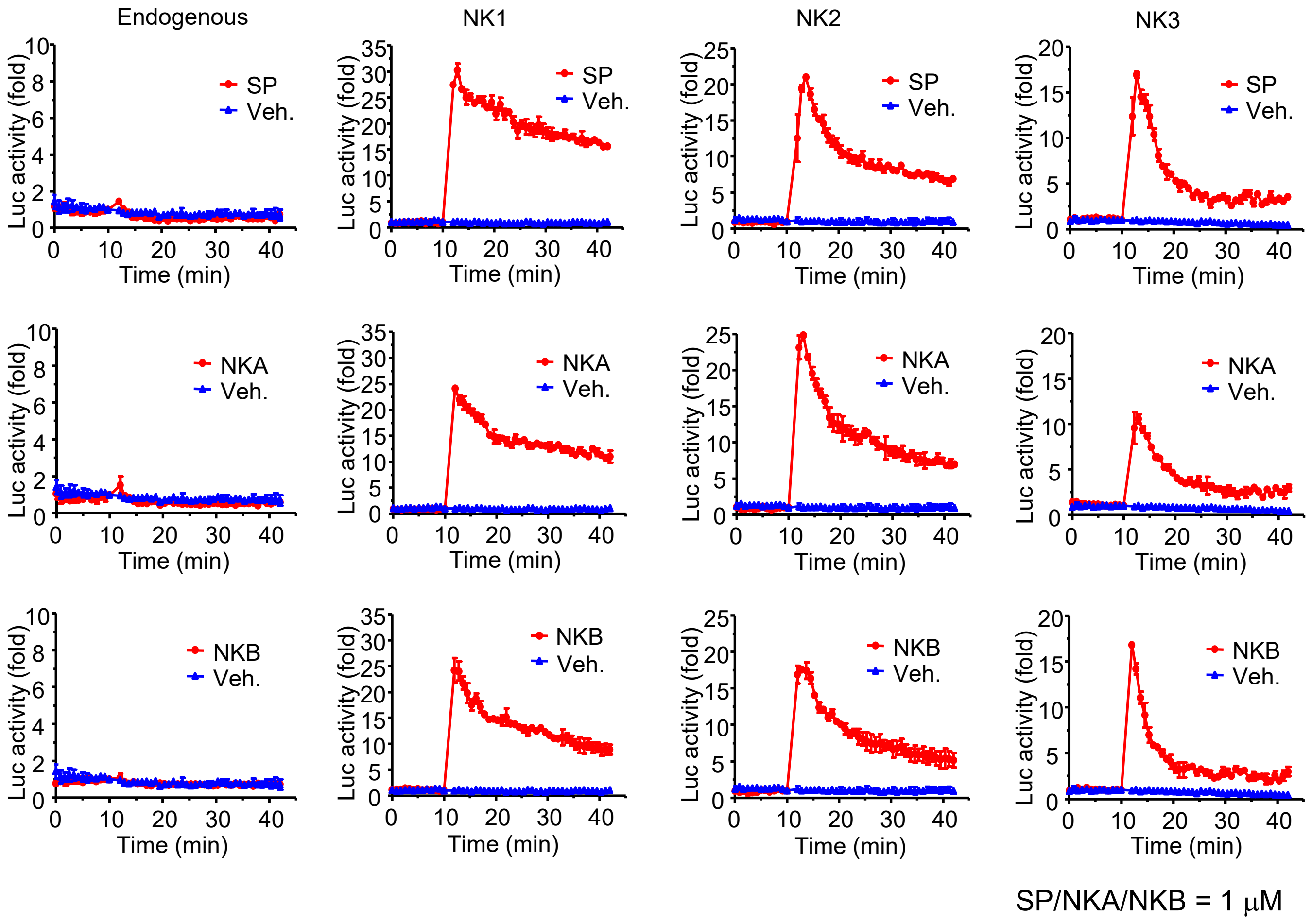
Endogenous
NK1
NK2
NK3
SP
Veh.
NKA
Veh.
NKB
Veh.
Luc activity (fold)
Time (min)
SP/NKA/NKB = 1 μM

Sup. Fig. 1

A

Calcium response: Calmodulin-SmBiT / LgBiT-MYLK2S

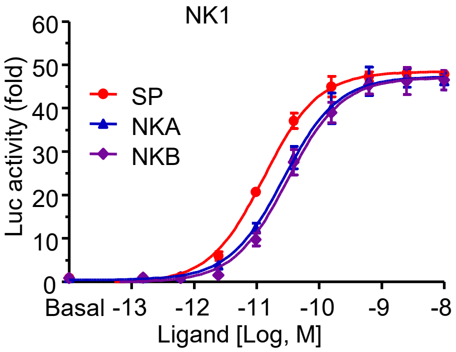

Ligand	EC_{50} (nM)
SP	0.013
NKA	0.027
NKB	0.031

Potency	SP>NKA=NKB
Efficacy	SP=NKA=NKB

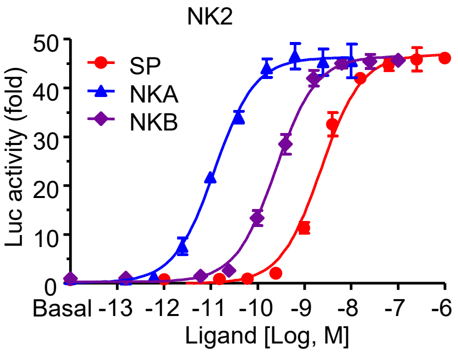

Ligand	EC_{50} (nM)
SP	2.200
NKA	0.012
NKB	0.240

Potency	NKA>>NKB>SP
Efficacy	SP=NKA=NKB

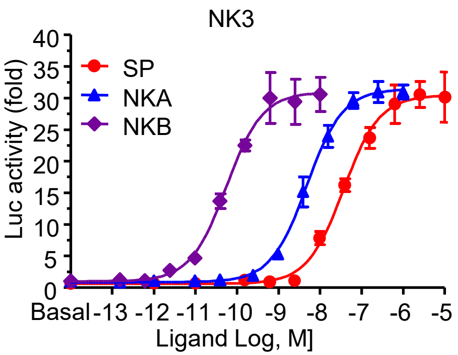

Ligand	EC_{50} (nM)
SP	37.300
NKA	4.800
NKB	0.056

Potency	NKB>>NKA>SP
Efficacy	SP=NKA=NKB

B

β-arrestin recruitment

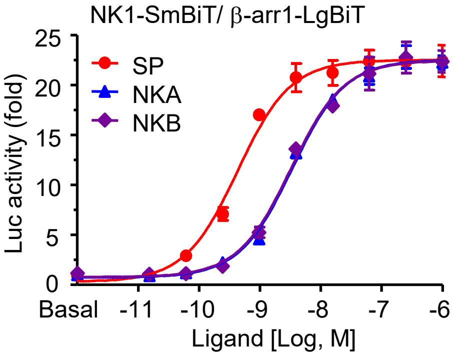

Ligand	EC_{50} (nM)
SP	0.41
NKA	3.42
NKB	3.21

Potency	SP>NKA=NKB
Efficacy	SP=NKA=NKB

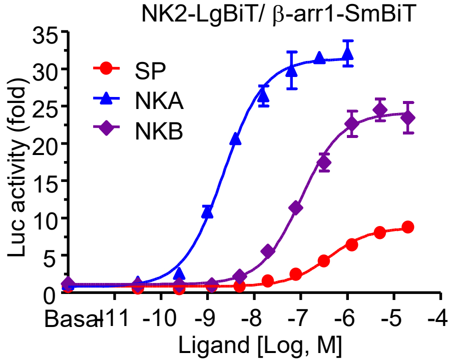

Ligand	EC_{50} (nM)
SP	409.0
NKA	2.2
NKB	101.0

Potency	NKA>>NKB>SP
Efficacy	NKA>NKB>>SP

Sup. Fig. 2

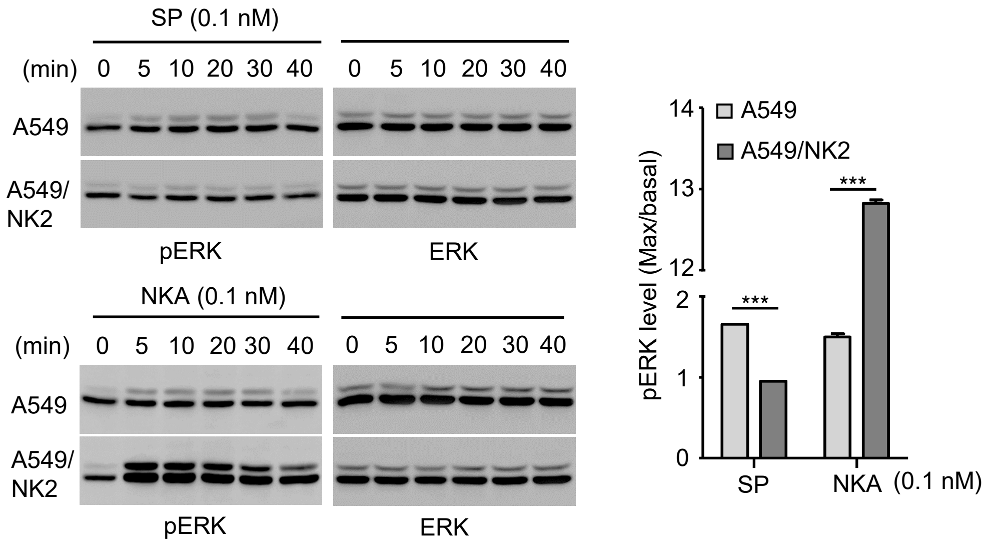
SP (0.1 nM)
(min) 0 5 10 20 30 40 0 5 10 20 30 40
A549
A549/ NK2
pERK
ERK
NKA (0.1 nM)
(min) 0 5 10 20 30 40 0 5 10 20 30 40
A549
A549/ NK2
pERK
ERK
A549
A549/NK2
pERK level (Max/basal)
14
13
12
2
1
0
***
***
SP
NKA
(0.1 nM)

Sup. Fig. 3

Supplementary information

raw data for Fig.3B

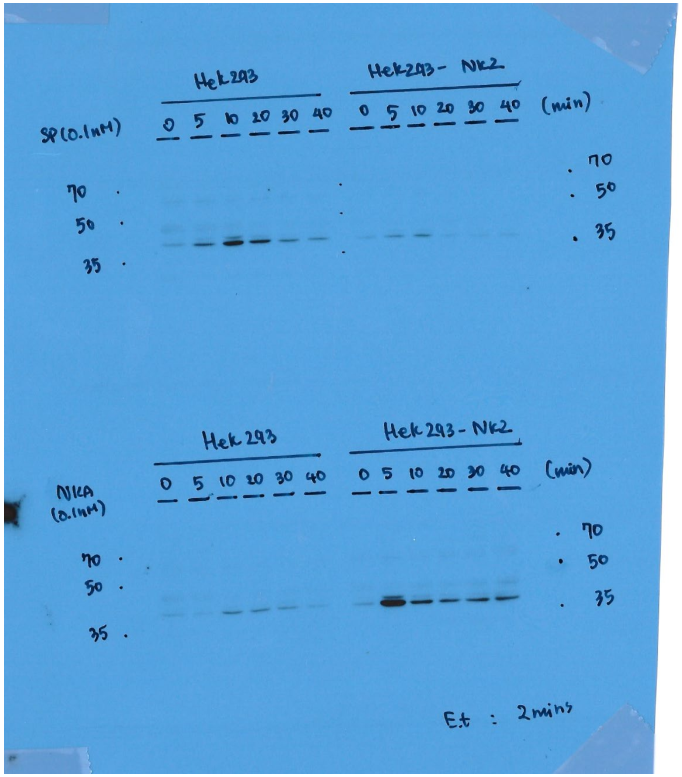

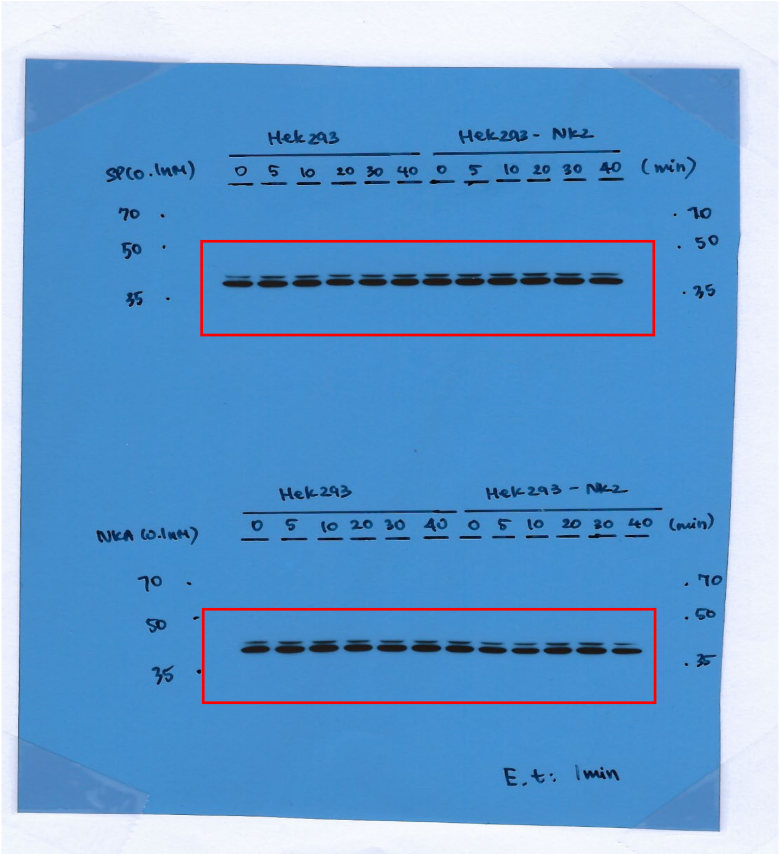

Supplementary information

raw data for Fig.5B

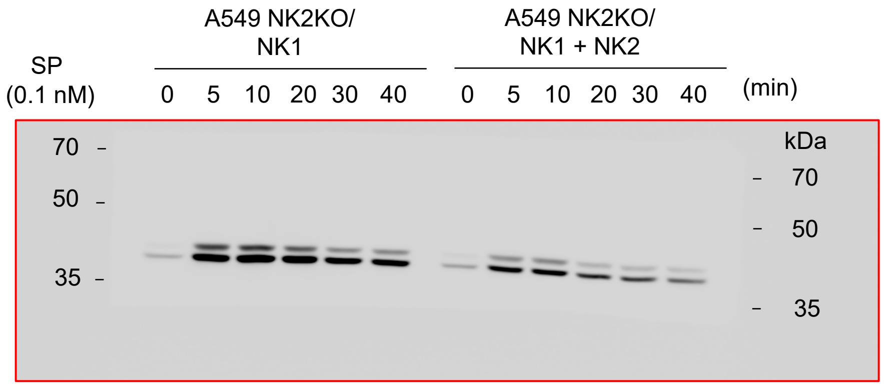

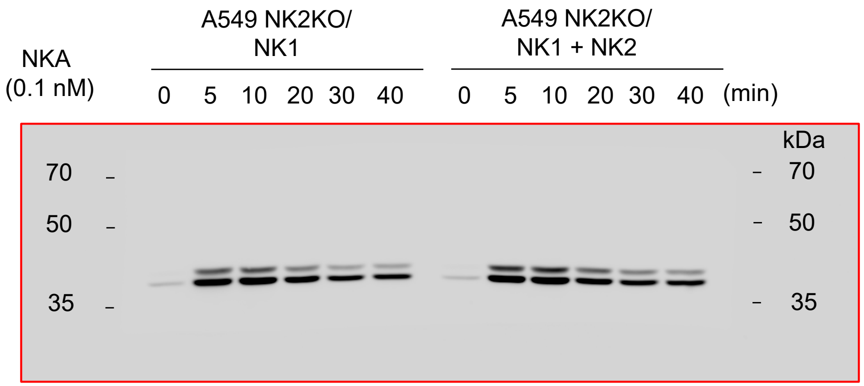

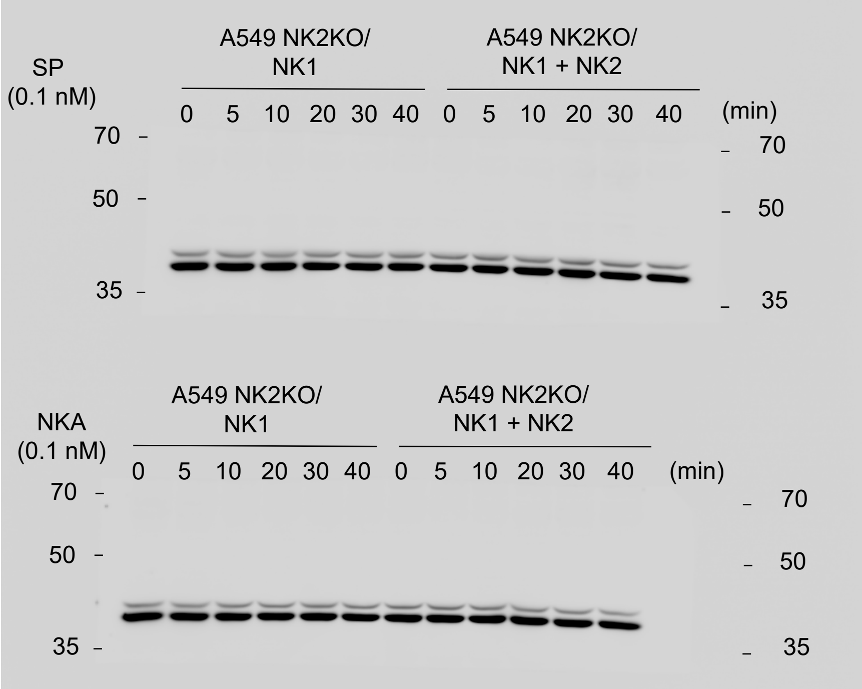

Supplementary information

raw data for supplementary Fig.3

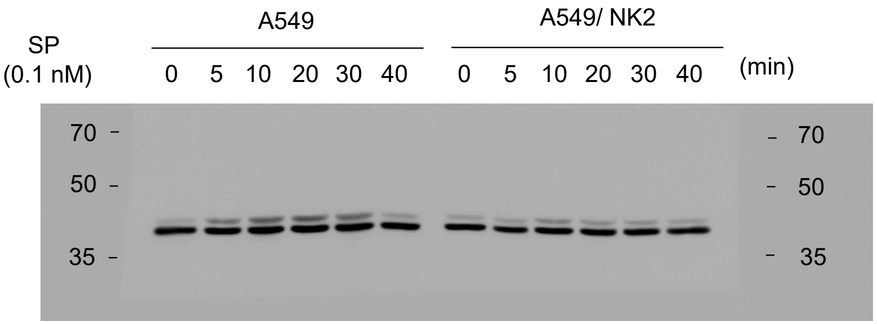

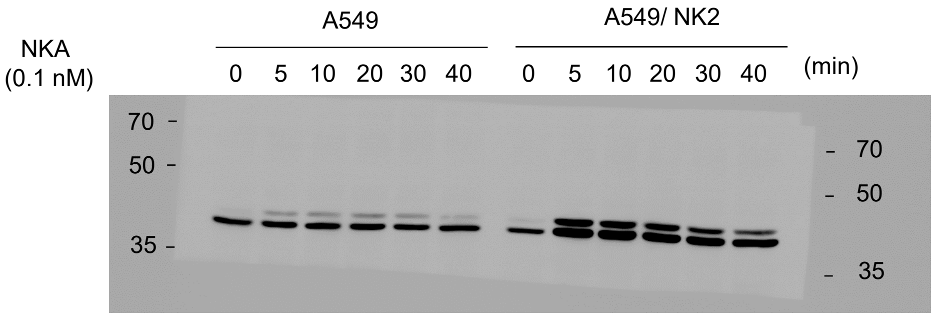

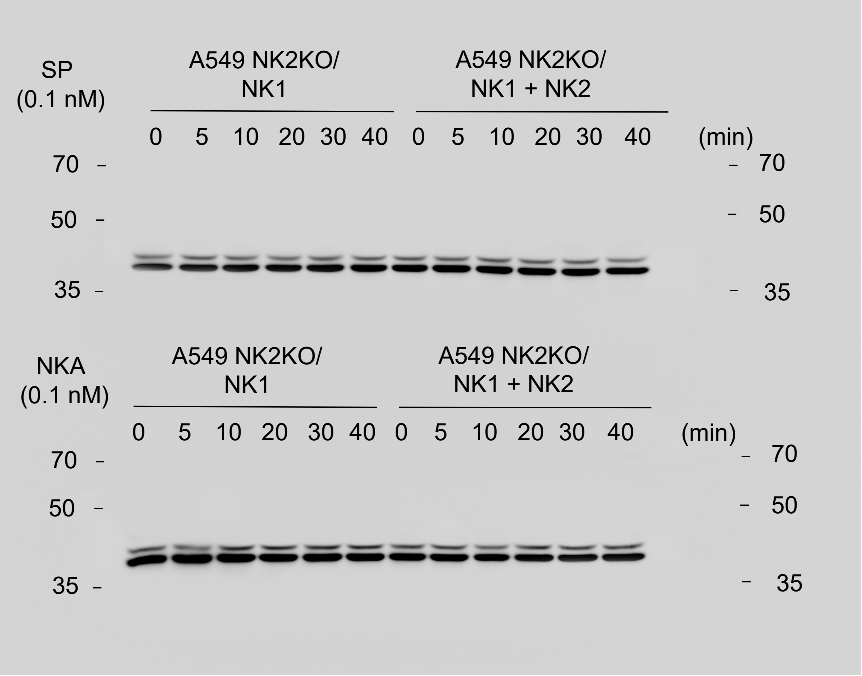

Supplement: Supplementary file 1 — Additional file 1: Fig. S1 Ca2+ responses mediated by all neurokinin receptors (NKs) to 1 μM tachykinins. HEK293 cells expressing Ca2+ probes with each NK were treated with 1 μM of SP, NKA, and NKB. Luciferase activities were measured with a luminometer. Fig. S2 Dose responses of NKs to each tachykinin. A HEK293 cells expressing Ca2+ probes with each NK were treated with serially dilute ed ligands and luciferase activities were measured. B Cells expressing NanoBiT constructs of β-arrestin1 and NKs were treated with serially diluted ligands. Their EC50 values were designated in the tables. Fig. S3 The effect of exogenous NK2 on tachykinin-stimulated ERK phosphorylation. Parental A549 cells and exogenous NK2-expressing cells were incubated with serum-free media for 24 h and treated with 0.1 nM tachykinins for designated time. After lysis the cell extracts were applied to western blotting with anti-ERK and anti-pERK antibodies. The graph shows maximal pERK levels in comparison to basal levels, which were normalized with ERK blots. ***: p<0.001 vs maximal pERK in parental cells. [file 13578_2023_1165_MOESM1_ESM.pdf]
